# Age and sex-related differences in elastic properties of the gastrocnemius muscle-tendon unit: an observational prospective study

**DOI:** 10.3389/fragi.2024.1455404

**Published:** 2024-11-11

**Authors:** Zhijie Zhang, Wenjing Wang, Feng Li, Jiayi Guo

**Affiliations:** ^1^ Rehabilitation Therapy Center, Luoyang Orthopedic Hospital of Henan Province, Orthopedic Hospital of Henan Province, Luoyang, China; ^2^ Department of Sport Rehabilitation, Shanghai University of Sport, Shanghai, China

**Keywords:** gastrocnemius, achilles tendon, elasticity imaging techniques, sex, aging

## Abstract

**Introduction:**

Changes in the mechanical properties of the gastrocnemius muscle-tendon unit can lead to abnormal biomechanics of lower limbs, which is a risk factor for the development of many diseases. However, fewer studies have explored physiological changes in the gastrocnemius muscle-tendon unit stiffness. This study aimed to investigate the age- and sex-related differences in the gastrocnemius muscle-tendon unit stiffness.

**Methods:**

The study included 20 older women, 20 young women, and 20 older men. Shear wave elastography (SWE) was used to measure the stiffness of the medial gastrocnemius (MG), lateral gastrocnemius (LG), and Achilles tendon (AT) in all subjects in relaxed, neutral, and standing positions.

**Results:**

The results showed no significant differences in the MG, LG, and AT stiffness between the dominant and non-dominant sides (*p* > 0.05). The MG, LG, and AT stiffness changed with positions (*p* < 0.05). The LG stiffness in older women was lower than in older men and young women in any position (*p* < 0.05). The MG stiffness in older men was greater than that in older women in any position, and age-related changes were found only in the relaxed and standing positions (*p* < 0.05). The AT stiffness was higher in older men only in the relaxed position (*p* < 0.05). There was no significant difference in AT stiffness between older and younger women at any position (*p* > 0.05).

**Discussion:**

These results suggest that the bilateral gastrocnemius muscle-tendon unit stiffnesses were similar regardless of sex, age, and position. The stiffness of the gastrocnemius in women decreased with age. However, the effect of aging on AT stiffness was slight. Men have greater gastrocnemius stiffness in older adults.

## 1 Introduction

Muscles are connected to the skeletal system via tendons to transfer power during movement (Finni, 2006). Tendons cushion the effects of mechanical loads to protect muscle fibers from injury (Griffiths, 1991). The muscle-tendon unit (MTU) saves energy by storing and releasing elastic energy during walking and running, particularly in the triceps surae and quadriceps (Sasaki and Neptune, 2006). The gastrocnemius MTU spans the knee and ankle joints and maintains gait stability. Numerous studies have suggested that the gastrocnemius MTU plays a vital role in the pedal phase of the gait cycle. The mechanical and morphological characteristics of MTUs are closely related to athletic performance. A large cross-sectional area results in excellent explosive power and endurance (Earp et al., 2010; Kovács et al., 2020). The stiffness of the gastrocnemius MTU positively correlated with jumping and sprinting (Ando et al., 2021; Kalinowski et al., 2022). Lower gastrocnemius muscle (GM) and Achilles tendon (AT) stiffness negatively affects balance control during walking. However, excessive stiffness of the gastrocnemius MTU may reduce joint flexibility and increase the risk of sports injuries (Gleim and McHugh, 1997). The gastrocnemius MTU improves sports outcomes during jump and landing by active contraction and passive stretching. Abnormal and repetitive movements are one of the biomechanical risk factors for soft tissue injury (Cocco et al., 2024). Numerous published studies have shown that abnormal increases and decreases in gastrocnemius MTU stiffness are closely related to disease occurrence (Monteagudo et al., 2018). Therefore, determining the stiffness of the gastrocnemius MTU in individuals with different demographic characteristics can identify physiological changes that can help in early disease prevention and diagnosis.

Physiological factors such as age and sex are related to muscle and tendon characteristics. Men have higher tissue cross-sectional area and strength, particularly in the upper limbs. The upper and lower limb muscle strengths in women are approximately 52% and 66%, respectively, of those in men (Miller et al., 1993; Bredella, 2017). MTU morphology changes and structural remodeling of the MTU with age. Aging leads to decreased volume and thickness of muscles and tendons, affecting mass and stiffness reduction (Wu et al., 2020). This change can be recognized after 50 years and is more pronounced in the soft tissues of the lower extremities (Janssen et al., 2000). The stiffness of the MTU is affected by changes in its morphology and composition. In older adults, the risk of falls is associated with GM thickness and stiffness, which is higher than with muscle mass (Kim et al., 2022). Some published papers have reported age- and sex-related differences in GM or AT stiffness. Hirata et al. reported that GM stiffness in older men was significantly lower than in younger men, at plantarflexion 30° and neutral position, with no difference at the maximum dorsiflexion angle (Hirata et al., 2020). A recent study showed that lateral gastrocnemius (LG) stiffness was similar between the ages of 20–40 and 60–69 years, but significantly decreased after 70 years of age (Pang et al., 2021). The changes in AT with age remain controversial (Turan et al., 2015). However, these studies explored age-related differences focused on men or did not distinguish between men and women. Few studies have explored changes in the gastrocnemius MTU in women by aging alone. Moreover, higher gastrocnemius MTU stiffness has been demonstrated in young men but less explored in the elderly (Taş and Salkın, 2019). Changes in gastrocnemius MTU stiffness with aging between men and women may be inconsistent owing to hormones. Hence, exploring the trend of gastrocnemius MTU stiffness with age in women and sex-related differences in older adults is necessary. In addition, previous studies suggested that the dominant side legs may bear more load in athletes (Leung et al., 2017). It is meaningful to explore and compare the effect of daily activities on the gastrocnemius MTU stiffness between dominant and non-dominant sides in different healthy individuals.

Shear wave elastography (SWE) is a relatively new tool for quantifying the elastic properties of soft tissues. It is non-invasive and provides skeletal muscle and tendon stiffness in real time. Recently, SWE has been applied more frequently to evaluate musculoskeletal and nervous system diseases and has shown good validity and reliability (Peltz et al., 2013; Kozinc and Šarabon, 2020). SWE can identify early pathological tissues conducive to early intervention, accurate rehabilitation, and accurate treatment. Previous studies found increased stiffness of the gastrocnemius MTU in individuals with plantar fasciitis and decreased tendon stiffness in patients with Achilles tendinopathy using SWE (Gatz et al., 2020; Zhou et al., 2020; Pan et al., 2021). This study evaluated the gastrocnemius MTU stiffness in different age and sex groups at three positions and compared the stiffness between dominant and non-dominant sides by SWE, which provides support for the physiological changes of tissue mechanical properties and helps researchers reduce the negative impact of physiological factors on the results during the experiment. Moreover, this study lays a foundation for future research to recognize pathological stiffness in soft tissue by SWE.

The purpose of this study was to explore: 1) the symmetry of the medial gastrocnemius (MG), LG, and AT stiffness between the dominant and non-dominant sides; 2) the stiffness of the LG, MG, and AT in relaxed, neutral, and standing positions; 3) the changes in LG, MG, and AT stiffness with age in women; and 4) the sex-related changes in LG, MG, and AT stiffness in older adults.

## 2 Methods

### 2.1 Subjects

Sixty subjects (20 older men, 20 older women, and 20 young women) volunteered to participate in the study from March 2023 to May 2023 (Table 1). This study was conducted at the Rehabilitation Therapy Center of Luoyang Orthopedic Hospital of Henan Province. The inclusion criteria were as follows: 1) the older men and women aged >50 years and young women aged 18–30 (Janssen et al., 2000); 2) no musculoskeletal or neurological disorders; 3) no surgery or injury history to the lower limbs; and 4) no skin lesions in the measurement regions. The exclusion criteria were as follows: 1) physical activity for more than 30 min in the 48 h before the start of the experiment; 2) severe cognitive impairment or mental illness; and 3) acute, infectious, infectious diseases or visceral insufficiency. This study was approved by the Ethics Committee of Luoyang Orthopedic Hospital of Henan Province (KY 2023-008–01). The study adhered to the principles of the Declaration of Helsinki. All participants voluntarily signed an informed consent form before the experiment.

**TABLE 1:** The characteristics of the subjects (mean ± SD).

	Older men	Older women	Young women
Age, years	58.40 ± 3.69	59.90 ± 5.84	22.30 ± 1.75
Height, m	1.74 ± 0.05	1.61 ± 0.04	1.61 ± 0.06
Weight, kg	71.59 ± 8.27	59.41 ± 7.20	55.20 ± 6.10
BMI, kg/m^2^	23.56 ± 2.30	22.99 ± 2.13	21.23 ± 1.98

### 2.2 Equipment

An ultrasonographic device (Aixplorer Supersonic Imagine, France) with a 4–15 MHz linear transducer array (SL15-4) was used to quantify the stiffness of the GM and AT. The ultrasonic transducer quickly emits sound radiation, generates transverse shear waves inside the tissue, and dynamically presents an image in real time (Ryu and Jeong, 2017). The shear wave speed (m/s) can be converted into shear modulus (kPa) as follows (Sigrist et al., 2017):

$$\mu =\rho c^{2}$$

where μ is the shear modulus, ρ is the material density, and c is the wave speed. Shear modulus is highly linearly related to elastic modulus and can be used as a substitute for stiffness when the transducer is parallel to the tissue fiber (Eby et al., 2013). A higher value indicates stiffer tissues, as shown in red. Blue indicates a lower stiffness (Ferraioli et al., 2012). The parameters were set to the musculoskeletal (MSK) mode, the depth of B-scan ultrasound was 4.0 cm, the image display was 50% opacity, and the measurement range from 0 to 600 kPa. The region of interest (ROI) was 4 mm, not covering tissues other than the GM or AT. The stiffness recorded on the image was the average of the ROI.

**FIGURE 1:**
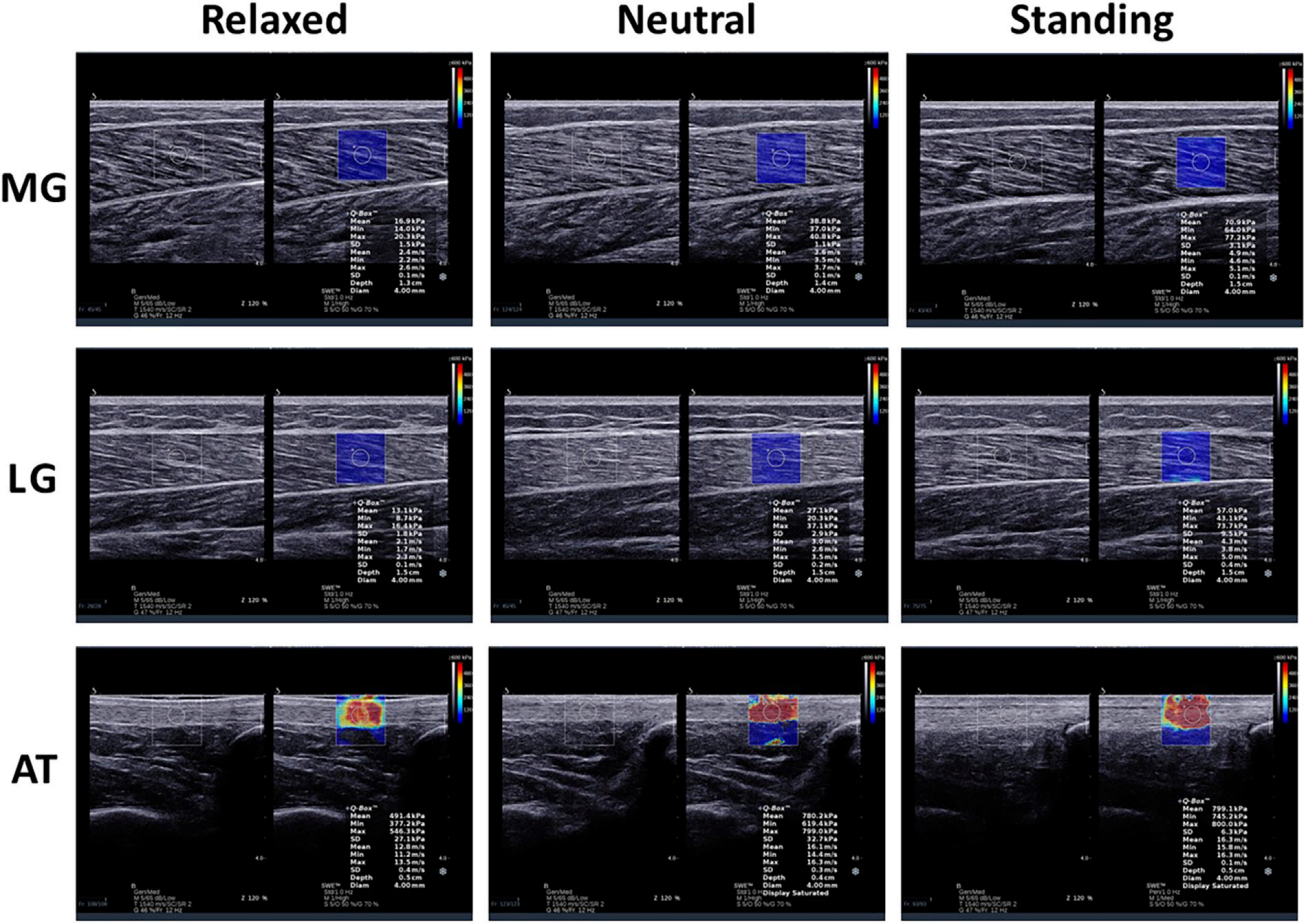
Longitudinal shear wave elastic imaging and grey scale sonogram of the medial gastrocnemius (MG), lateral gastrocnemius (LG), and Achilles tendon (AT) in different positions.

Moreover, a large amount of water-soluble coupler was used between the transducer and skin to avoid air interference with the image during the measurement. The transducer was placed perpendicular to the skin surface and longitudinally parallel to the muscle fibers. The operator ensured the transducer was free of pressure on the tissue during scanning. After recognizing this issue, the transducer remained for more than 5 s until the picture was sufficiently clear. This study obtained MG, LG, and AT elastic images of all subjects in the three positions (Figure 1).

### 2.3 Measurement of gastrocnemius and achilles tendon

The MG, LG, and AT stiffness were quantitatively measured using SWE. The MG stiffness was measured proximal to 30% of the length from the medial popliteal fossa to the lateral malleolus. The LG was estimated to be the proximal 30% length from the lateral popliteal fossa to the medial malleolus (Zhou et al., 2019). The cross-sectional areas of the MG and LG were maximal at these two sites. The AT stiffness was measured by placing the transducer at 4 cm above the calcaneal tubercle. AT injuries tend to occur 2–6 cm above the calcaneal tubercle. Before the experiment, a marker was used to mark the measurement site to ensure each measurement was in the same position and avoid measurement errors. The measurements were repeated three times for each tissue, and the average was calculated.

### 2.4 Procedures

Demographic characteristics such as sex, age, height, and weight were collected before the experiment. The room temperature was kept at 25°C throughout the experiment. The MG, LG, and AT stiffness on both sides of all subjects was measured sequentially in the relaxed, neutral, and standing positions. First, the subjects were asked to lie prone on the treatment bed with their hips and knees fully extended and their feet naturally placed on the outer edge of the bed. The MG, LG, and AT stiffness were measured in the relaxed position. The ankle was then fixed at 90° with a brace, and the stiffness of the muscles and tendons in the neutral position was estimated. During the measurement, the subjects were asked to avoid active muscle contractions in relaxed and neutral positions. Finally, the MG, LG, and AT stiffness in the standing position were measured as quickly as possible based on image stabilization to minimize the additional effects of time and changeful muscle contractions. The stiffness measured on the dominant side was selected for the analysis. The dominant side was determined as the subject used when kicking a ball (Lenskjold et al., 2015).

### 2.5 Reliability tests

Twelve subjects were randomly selected for the intra-observer reliability tests. Operator A repeatedly measured the MG, LG, and AT stiffness on the dominant side twice at different positions. The interval between the two measurements was 5 days. The results of the first measurement were used for the subsequent statistical analyses.

### 2.6 Statistical analysis

SPSS 26.0 software (version 26.0, IBM, United States) was used for the statistical analysis. Descriptive statistics were used to analyze the demographic characteristics and measured variables of the subjects. All statistical data are presented as mean ± standard deviation (SD). The normality of all data was analyzed using the Shapiro-Wilk test. Levene’s test was used to assess the homogeneity of variance. A non-parametric test was used to analyze the data when continuous variables were non-normal or showed heterogeneity of variance. The reliability of the muscle and tendon measurements at different positions was assessed using intraclass correlation coefficients (ICC). An ICC above 0.75 indicates excellent reliability; less than 0.40 means poor. A matched-sample t-test was used to compare the MG, LG, and AT stiffness between the dominant and non-dominant sides. Changes in stiffness with age and sex were assessed using an independent sample t-test. Changes in position were analyzed using a one-way analysis of variance (ANOVA). The Bonferroni correction was used for multiple comparisons when there were significant differences. *p* < 0.05 indicated that the difference was statistically significant.

## 3 Results

### 3.1 Reliability

The intra-observer reliabilities of the MG, LG, and AT measurements on the dominant side at different positions were excellent (Table 2). The ICC values for the MG ranged from 0.909 to 0.941 (relaxed: ICC = 0.931; neutral: ICC = 0.941; standing: ICC = 0.909). The ICC values for the LG ranged from 0.841 to 0.887 (relaxed: ICC = 0.886; neutral: ICC = 0.887; standing: ICC = 0.841). The ICC values for AT ranged from 0.893 to 0.978 (relaxed, ICC = 0.978; neutral, ICC = 0.945; and standing, ICC = 0.893). The intra-observer reliability of the LG in any position was lower than that of the MG and AT. Inner confidence was lowest in the standing position.

**TABLE 2:** Intra-observer reliability of stiffness measurements.

	Relaxed		Neutral		Standing	
	ICC	95%CI	ICC	95%CI	ICC	95%CI
MG	0.931	0.778–0.980	0.941	0.809–0.983	0.909	0.721–0.973
LG	0.886	0.653–0.966	0.887	0.668–0.966	0.841	0.469–0.954
AT	0.978	0.928–0.993	0.945	0.826–0.984	0.893	0.685–0.968

MG, medial gastrocnemius; LG, lateral gastrocnemius; AT, achilles tendon; ICC, intraclass correlation coefficient; CI, confidence intervals.

### 3.2 Dominant and non-dominant

Table 3 presents the stiffness of the dominant and non-dominant sides. There were no significant differences in the stiffness of the MG, LG, and AT between the dominant and non-dominant sides in any position, regardless of age and sex (*p* > 0.05).

**TABLE 3:** The stiffness of the dominant and non-dominant side in three positions for different groups (mean ± SD).

	Older men					Older women					Young women				
	Dominant	Non-dominant	P			Dominant			Non-dominant	P	Dominant		Non-dominant		P
Relaxed															
MG	18.21 ± 2.06	18.12 ± 2.34	0.807			15.04 ± 1.34			15.12 ± 1.05	0.695	16.83 ± 1.16		16.98 ± 1.08		0.579
LG	13.99 ± 1.43	14.00 ± 1.56	0.978			12.56 ± 1.33			12.42 ± 1.41	0.603	13.80 ± 1.24		13.91 ± 1.64		0.531
AT	584.04 ± 119.76	573.30 ± 124.16	0.296			510.96 ± 79.16			513.67 ± 82.73	0.749	516.80 ± 75.47		506.37 ± 77.75		0.395
Neutral															
MG	38.33 ± 5.26	37.64 ± 4.45	0.435			33.64 ± 3.67			33.74 ± 3.42	0.822	35.77 ± 3.96		36.88 ± 3.32		0.101
LG	27.11 ± 3.87	28.21 ± 4.71	0.206			23.01 ± 2.82			23.44 ± 2.76	0.411	27.36 ± 4.22		27.23 ± 3.62		0.862
AT	783.32 ± 23.41	785.37 ± 15.30	0.872			789.88 ± 18.46			789.51 ± 15.16	0.478	783.43 ± 21.53		784.60 ± 17.11		0.911
Standing															
MG	85.95 ± 15.77	88.44 ± 13.72	0.358			77.40 ± 8.92			78.64 ± 9.71	0.411	91.44 ± 12.25		88.55 ± 12.84		0.071
LG	55.30 ± 11.23	54.01 ± 10.24	0.526			46.03 ± 6.18			47.51 ± 6.37	0.442	54.90 ± 7.77		53.86 ± 7.84		0.485
AT	795.53 ± 5.28	793.18 ± 8.81	0.126			793.89 ± 6.87			794.28 ± 6.09	0.911	790.52 ± 10.44		787.03 ± 15.26		0.296

MG, medial gastrocnemius; LG, lateral gastrocnemius; AT, achilles tendon.

### 3.3 The stiffness of MG, LG and AT changed with sex and age

**FIGURE 2:**
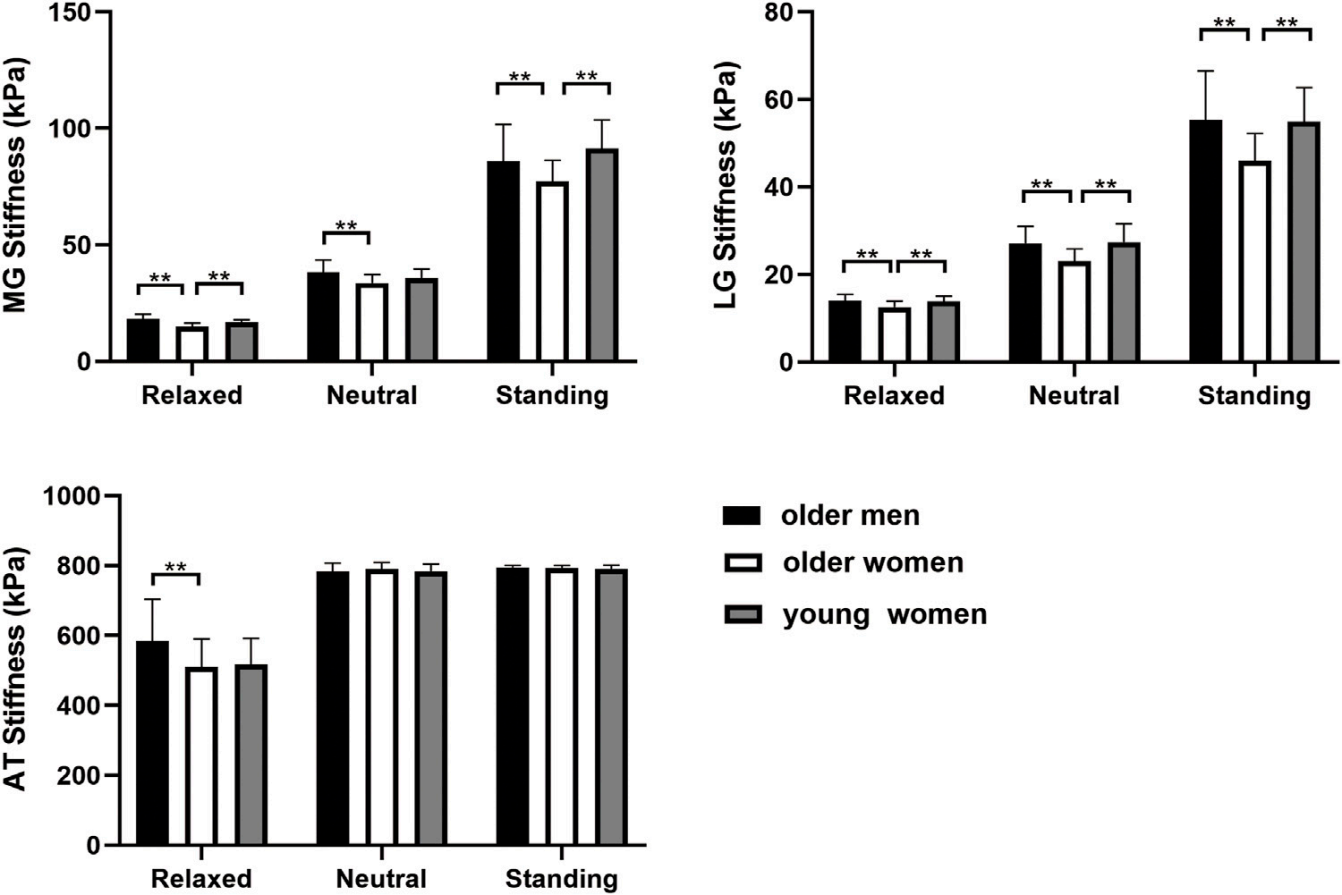
The stiffness of MG, LG, and AT within different age and sex groups in three positions. **p < 0.05.

Sex- and age-related changes in the MG, LG, and AT are shown in Figure 2. The stiffness of the MG was higher in older men than in older women in all positions (*p* < 0.05). The MG stiffness of the older women was lower than that of the young women in the relaxed and standing positions (*p* < 0.001). However, there was no significant difference in the neutral position (*p* > 0.05). The stiffness of the LG was significantly lower in older women than in young women and older men in all positions (*p* < 0.05). There was no significant difference in the AT stiffness between older and young women (*p* > 0.05). The stiffness of the AT in older women was not significantly different from that in older men, except in the relaxed position (*p* > 0.05).

### 3.4 The stiffness of MG, LG and AT changed with positions

**FIGURE 3:**
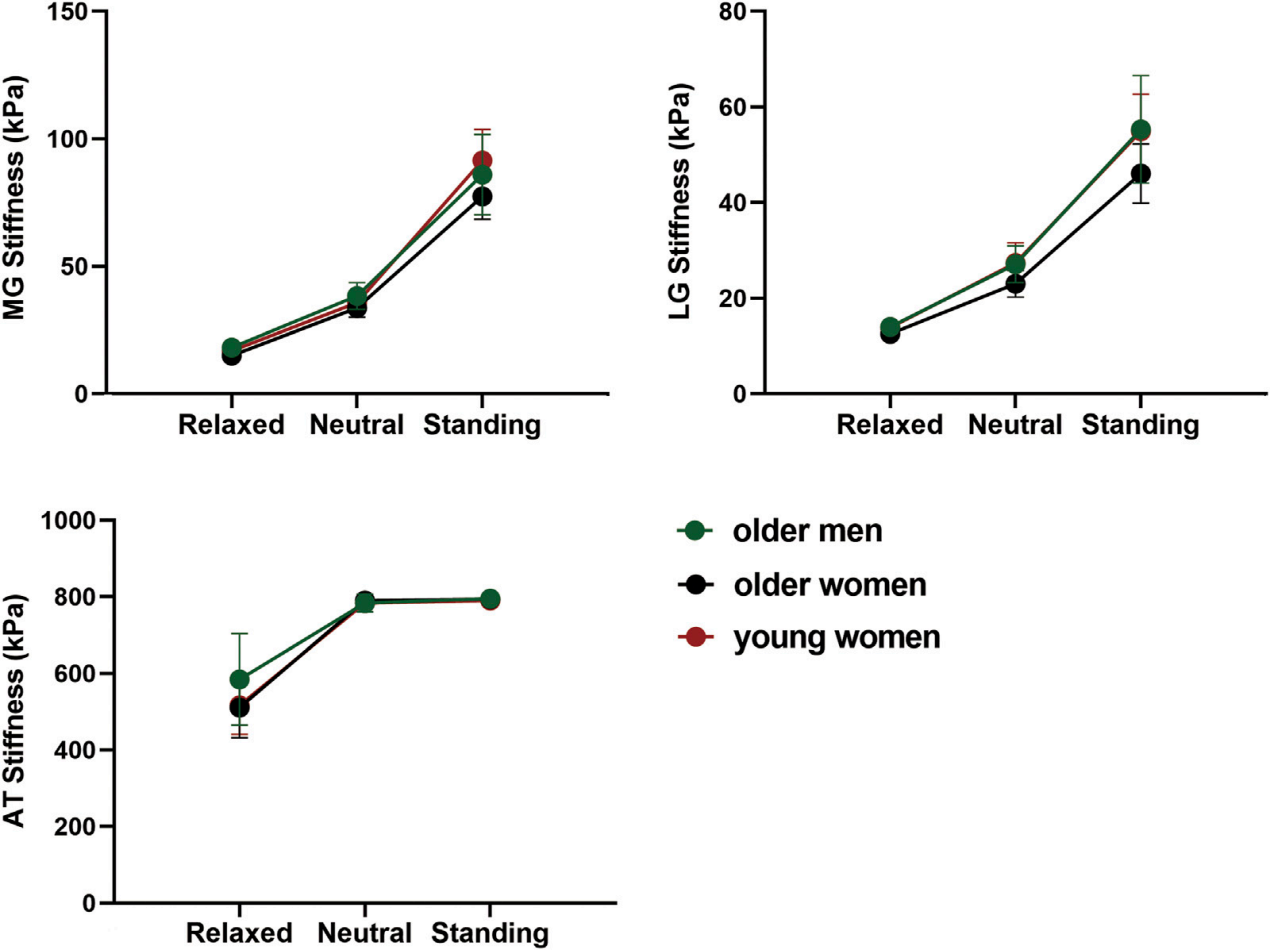
The variation trend of MG, LG, and AT stiffness with position in different age and sex groups.

Figure 3 shows the stiffness changes of MG, LG, and AT in different positions. The stiffness of the GM and AT were highest in the standing position and lowest in the relaxed position (*p* < 0.05). The stiffness of the MG was higher than that of the LG, and that of the AT was the highest in all positions.

## 4 Discussion

This study used SWE to evaluate the bilateral gastrocnemius-Achilles tendon complex stiffness in young women, older men, and older women in different positions. Three main findings emerged from the study: (1) the stiffness of the MG, LG, and AT were similar on the dominant and non-dominant sides regardless of position, sex, and age; (2) the stiffness of the gastrocnemius in older men was higher than that in older women, and AT was higher only in the relaxed position; and (3) compared with young women, the stiffness of the gastrocnemius in older women was lower in the relaxed and standing positions, and there was no significant difference in AT. Previous studies have explored age- and sex-related differences in muscle stiffness, but few have evaluated sex differences in stiffness in older adults or age-related changes in women.

This study found that the stiffness of the gastrocnemius-Achilles tendon complex was not affected by the dominant side, and the findings were shown in different groups at the three positions. Age, sex, and position appeared to affect the MG, LG symmetrically, and AT stiffness in both lower limbs. These results suggest that the stiffness of the MG, LG, and AT measured on either side may not consider the additional effects caused by the dominant side in future studies. A previous study showed that the MG, LG, and AT stiffness between the dominant and non-dominant sides was not significantly different between amateur basketball players and non-athletes at both ankle neutral and 10° dorsiflexion, which was similar to the results of this study (Chang et al., 2020). However, the dominant leg may have to bear more load during certain activities. Long-term repeated mechanical loads increase the cross-sectional area and stiffness of the muscle-tendon unit (Leung et al., 2017). The stiffness of the AT is significantly higher on the dominant side than on the non-dominant side in jumping athletes, which may support a higher risk of injury on the dominant side (Bayliss et al., 2016). Long-term specific physical activities of subjects should be considered in future studies to prevent interference with experimental results when the non-dominant side is used as a control.

Leg muscle mass and strength decrease with aging (Delmonico et al., 2009). Skeletal muscle mass begins to decrease at thirty, and a significant reduction is observed at the age of 50 years, mainly attributable to the lower extremities (Janssen et al., 2000). Muscle stiffness is considerably correlated with strength and mass in all age groups (Alfuraih et al., 2019). This study observed that gastrocnemius stiffness in older women in relaxed and standing positions was lower than that in younger women. Previous studies have shown that the number and function of fibro-adipogenic progenitors change with aging, affecting muscle regeneration (Lai et al., 2024). The fibro adipose tissue presents a specific B-mode sonographic pattern with increased echogenicity, supporting the findings of reduced stiffness in older women compared to young women (Jacisko et al., 2023). Age-related changes in muscle characteristics are essential for reduced quality of life in older adults (Larsson et al., 2019). A previous study found that women aged >60 and men aged >50 had reduced GM thickness compared to adults aged approximately 20 (Fujiwara et al., 2010). Another study found that the GM had a significantly lower thickness and stiffness of relaxed and contracted structures in adults above 50 compared to 18–30 adults (the stiffness increase rate was similar), which is consistent with the results of this study (Şendur et al., 2020). However, a recent study showed that the contracted shear wave speed of the LG did not decrease significantly until 70 years of age, and the relaxed SWV did not change with age (Pang et al., 2021). The analysis results for the shear wave velocity and shear modulus may differ. Regarding sex-related differences, we observed higher GM stiffness in older men than in women. A previous study assessed the gastrocnemius MTU stiffness in 54 healthy people aged 19–27 years by Myoton PRO and found that MG stiffness was higher in young men than in women (Taş and Salkın, 2019). Skeletal muscle mass in men is significantly greater than in women. Sex differences in GM stiffness may be attributed to soft tissue structure and intrinsic composition.

There was no difference in the stiffness of the AT between age groups in this study. Another study also found that the shear wave speed of AT in the relaxed position did not change with age (Fu et al., 2016). However, a previous study observed that AT reduced stiffness and increased cross-sectional area (CSA) in adults aged 65–75 (Lindemann et al., 2020). The average age of the subjects in this study was 59 years, and AT stiffness may be in recession until 70. The published study reported that adults over 50 years also had an increased CSA of the AT, which may be a compensatory mechanism to offset the reduced muscle material properties (Pang and Ying, 2006). Loss of muscle quality occurs at different times (Do et al., 2021). The stiffness of the GM may decrease with age earlier than that of the AT. However, some studies have found increased AT stiffness in older adults, which may be associated with the risk of AT rupture (Turan et al., 2015; Slane et al., 2017). There are significant differences in the stiffness changes of the AT with age, which may be related to the different research subjects, equipment, and sites. Sex-related changes in AT stiffness were not observed in the neutral and standing positions. However, higher AT stiffness in young men than in young women has been observed at 0° and 10° of ankle joint dorsiflexion (Taş and Salkın, 2019). Age-related changes in muscle and tendon stiffness may differ between men and women, and may be related to hormone levels. Estrogen levels are significantly lower in postmenopausal women, leading to a decline in muscle mass and strength (Ikeda et al., 2019). Studies have shown that higher estrogen levels are associated with lower AT stiffness, possibly reducing the risk of AT injury (Chidi-Ogbolu and Baar, 2018). The risk of AT injury is lower in premenopausal women than men and is similar in postmenopausal women when the estrogen level is equivalent to that in men (Maffulli et al., 1999; Smith et al., 2008; Huttunen et al., 2014).

The tension of the relaxed fibers under a low load causes increased stiffness of the muscle-tendon tissue. The elastic properties of soft tissues play essential roles in their motion. The elastic potential energy is stored owing to the tensile load and released during muscle contraction to improve efficiency. Numerous studies have demonstrated increased stiffness of the GM and AT from ankle plantarflexion to dorsiflexion. However, studies on variations in AT and MG stiffness with ankle angle in older adults are lacking. This study found that the stiffness of the GM and AT were significantly higher in the neutral position than in the relaxed position in older adults. The degree of passive muscle elongation positively correlates with tissue stiffness in a particular range, regardless of age. In addition, we evaluated GM and AT stiffness in the standing position. The stiffness of the GM increased significantly in the standing position. Standing did not seem to lead to a higher AT stiffness. The GM significantly contributes to bearing weight load and maintaining joint stability in the standing position. The morphology of the GM has a stronger correlation with functional motor ability, flexibility, and balance than the AT in older women (Muanjai et al., 2022). The shift in stiffness with position differed between the GM and AT. Sex - and age-related stiffness changes differed among tissues at the three positions. The inconsistent results between the GM and AT may be related to the unique biological characteristics of the muscles and tendons.

This study had some limitations. First, we did not assess soleus stiffness in different positions because the technical limitations of the deep muscles could not be identified. Second, GM and AT stiffness measurements in the neutral and standing positions may have been affected by time, although we measured them as quickly as possible. Third, no electromyography was used to monitor muscle activity during the experiment. Active muscle contractions may still occur, although the subjects were asked to avoid them in the prone position as much as possible. Additionally, we could not exclude the effects of daily physical activity levels on muscle and tendon stiffness, which were not documented in this study. Finally, no young men were included. Future research should explore sex differences in age-related changes in GM and AT stiffness.

## Data Availability

The raw data supporting the conclusions of this article will be made available by the authors, without undue reservation.
